# Climate change and women’s cancer in the MENA region: assessing temperature-related health impacts

**DOI:** 10.3389/fpubh.2025.1529706

**Published:** 2025-05-27

**Authors:** Wafa Abu El Kheir-Mataria, Sungsoo Chun

**Affiliations:** Institute of Global Health and Human Ecology, American University in Cairo, Cairo, Egypt

**Keywords:** women’s health, MENA region, ambient temperature, cancer prevalence and mortality, climate change and health

## Abstract

**Introduction:**

Climate change poses a significant threat to public health, exacerbating health inequalities. Women in the Middle East and North Africa (MENA) region, identified as high-risk, are particularly affected.

**Objective:**

This study investigates the influence of rising temperatures on cancer prevalence and mortality among women in the MENA region, filling critical knowledge gaps.

**Methods:**

We employed Multiple Linear Regression (MLR) analysis to examine the correlation between increased ambient temperatures and the prevalence and mortality of four types of cancer (breast, cervical, ovarian, and uterine) across 17 MENA countries.

**Results:**

Our analysis indicates a significant correlation between prolonged exposure to high ambient temperatures and all four cancer types studied. Notably, the prevalence of breast, ovarian, and cervical cancers is markedly influenced by temperature increases.

**Conclusion:**

The findings underscore the necessity of incorporating climate change adaptation strategies into national cancer control plans. Such integration is vital to mitigate the health impacts of climate change on women’s cancer prevalence and mortality in the MENA region.

## Background

Climate change is a global dilemma (1–3) affecting all aspects of life including health (4). Climate change results in rising temperatures, reduced air quality, threatened water supplies and safety, and increased food insecurity, which ultimately impact the health of populations (5–8). Rising temperatures contribute to the creation of favorable circumstances for disease vector breeding and illness dissemination and thus increase the risk of infectious diseases (9). Moreover, climate disasters disrupt a nation’s infrastructure, particularly its health systems, which challenges and reduces access to health care.

Cancer is a leading cause of death among women; in 2020, 4.4 million women worldwide succumbed to the disease, with 25% of these deaths attributed to breast cancer (10). Climate change exacerbates this burden by increasing exposure to carcinogenic risk factors. For instance, rising temperatures and environmental changes can elevate exposure to air pollutants, ultraviolet (UV) radiation, and endocrine-disrupting chemicals, all linked to higher cancer incidence (11). To understand how these environmental exposures interact with social and biological factors to influence health outcomes, we can apply the Eco-Social Theory, proposed by Nancy Krieger. This theory emphasizes the importance of considering the complex interplay between ecological systems and social structures in determining disease distribution (12).

Additionally, climate change can disrupt healthcare delivery systems, hindering timely cancer screening, diagnosis, and treatment, thereby adversely affecting cancer outcomes (13). Women, in particular, face heightened exposure to modifiable risk factors intensified by climate change, such as increased contact with environmental toxins and prolonged UV radiation exposure (14). Addressing these modifiable risk factors and strengthening healthcare infrastructure are crucial in mitigating the impact of climate change on women’s cancer risk.

Climate change disproportionately harms women due to social factors, such as cultural norms and economic disparities (15). The Health Equity Framework highlights how these disparities in economic power, healthcare access, and social structures create vulnerabilities, making it more challenging for women—especially in low-resource settings—to effectively mitigate cancer risks (16).

Additionally, physiological vulnerabilities, including pregnancy-related health risks, make women more susceptible to climate-related health impacts. Rising temperatures and exposure to particulate matter (PM) from air pollution have also been linked to increased morbidity and mortality in women. Furthermore, pregnant women are more susceptible to climate change. Pregnancy-related hazards, such as preterm birth, fetal development retardation, and hypertensive disorders, are increased (17).

Gender disparities, sociocultural norms, and historical inequalities. In the MENA region, gender disparities, sociocultural norms, and historical inequalities significantly impact women’s cancer outcomes. Limited healthcare autonomy, economic inequalities, and restrictive laws delay diagnoses and hinder timely treatment (18). In many societies, cultural taboos around reproductive and breast health reduce awareness and participation in cancer screening programs, while stigma surrounding gynecological cancers further discourages early medical intervention (19, 20).

The MENA region is particularly at risk due to global warming (21). By 2050, a temperature rise of 4°C is expected (22), with a yearly increase in drought incidence (23), making it “the most water-stressed region on Earth” (24). MENA region has a population of 493,264,873 with 48.3% females (25). In 2019, 420,812 people died from cancer in the region, 175,707 of whom were women (26). Given the region’s extreme climate conditions, women’s cancer risks may be further compounded by rising temperatures, worsening air pollution, water insecurity, and disruptions in healthcare systems, all of which can influence carcinogen exposure, cancer screening, and outcomes (13).

An eco-social perspective reveals how climate change amplifies environmental exposures contributing to cancer risk, while the health equity framework highlights its unequal burden on marginalized women with limited healthcare access. Sociological insights further underscore the role of gender norms, cultural restrictions, and historical inequalities in shaping cancer disparities in the MENA region.

Despite the growing body of research on the relation between climate change and women’s cancer, there are still many gaps in our knowledge. The current study aims at investigating the relationship between climate change and cancer in women in the MENA region by assessing the influence of rising ambient temperature on the prevalence and deaths caused by four types of women’s cancer: breast cancer, cervical cancer, ovarian cancer, and uterine cancer.

## Methods

### Research design

The study applies quantitative methods using Multiple Linear Regression (MLR) to analyze the correlation between rising ambient temperatures and female cancer burden using a two-level approach. First, an overall regional model (Table 1) was constructed using aggregated data from all 17 MENA countries to assess broader temperature trends in relation to four cancers prevalence and mortality in women across the region.

**Table 1:** Correlation between women cancers prevalence and deaths and temperature.

Dependent variable		Independent variable	Standard error (SE)	B coefficient	*t*	*p*	Model summary
Prevalence in females (Percentage)	Ovarian cancer	Temperature	0.000	0.280	5.564	0.000	R^2^ = 0.08; F = 15.962 *p* < 0.000
		GDP	0.000	−0.083	−1.657	0.98	
	Cervical cancer	Temperature	0.000	0.207	4.038	0.000	R^2^ = 0.058; F = 11.310 *p* < 0.000
		GDP	0.000	−0.151	−2.979	0.003	
	Breast cancer	Temperature	0.000	0.173	3.338	0.001	R^2^ = 0.030; F = 5.595 *p* < 0.04
		GDP	0.000	−0.032	−0.620	0.536	
	Uterine cancer	Temperature	0.000	0.220	4.304	0.000	R^2^ = 0.000; F = 0.005 *p* < 0.995
		GDP	0.000	−0.099	−1.929	0.054	
Deaths in females (Percentage)	Ovarian cancer	Temperature	0.000	0.332	6.755	0.000	R^2^ = 0.123; F = 25.754 *p* < 0.000
		GDP	0.000	−0.159	−3.234	0.001	
	Cervical cancer	Temperature	0.000	0.171	3.344	0.001	R^2^ = 0.055; F = 10.752 *p* < 0.000
		GDP	0.000	−0.184	−3.598	0.000	
	Breast cancer	Temperature	0.002	0.269	5.411	0.000	R^2^ = 0.102; F = 20.974 *p* < 0.000
		GDP	0.000	−0.209	−4.196	0.000	
	Uterine cancer	Temperature	0.000	0.213	4.243	0.000	R^2^ = 0.083; F = 16.610 *p* < 0.000
		GDP	0.000	−0.221	−4.392	0.000	

Second, for each cancer type prevalence and mortality, MLR analysis was done on the disaggregated data (Tables 2–5) to examine these correlations at the national level, allowing for a more detailed analysis of temperature variations across countries. To account for socioeconomic disparities that may influence health outcomes, GDP per capita was included as a control variable. This two-tiered approach provides a more focused assessment of how rising temperatures correlate with female cancer outcomes while reducing potential socioeconomic confounding factors.

**Table 2:** The correlation between BCP/BCD and TEMP for each country.

Country	Breast cancer prevalence (BCP)							Breast cancer death (BCD)						
	R square	F	*p*	independent variable	Beta	*t*	*p*	R square	F	*p*	independent variable	Beta	*t*	*p*
1	0.873	65.497	0.000	Temp	−0.030	−0.366	0.719	0.966	271.018	0	Temp	−0.012	−0.287	0.777
				GDP	0.937	11.411	0.000				GDP	0.984	23.172	0
2	0.826	45.192	0.000	Temp	0.333	3.285	0.004	0.662	18.631	0	Temp	0.165	1.165	0.258
				GDP	0.743	7.326	0.000				GDP	0.744	5.266	0
3	0.910	C	0.000	Temp	−0.059	−0.709	0.487	0.589	13.591	0	Temp	−0.092	−0.518	0.61
				GDP	0.986	11.855	0.000				GDP	0.816	4.573	0
4	0.543	C	0.000	Temp	0.300	1.917	0.070	0.728	25.391	0	Temp	0.245	2.033	0.056
				GDP	0.635	4.062	0.001				GDP	0.786	6.509	0
5	0.626	15.906	0.000	Temp	0.111	0.717	0.482	0.663	18.726	0	Temp	0.13	0.891	0.384
				GDP	0.739	4.784	0.000				GDP	0.751	5.13	0
6	0.211	2.543	0.105	Temp	0.408	2.000	0.060	0.457	7.989	0.003	Temp	0.418	2.469	0.023
				GDP	0.216	1.060	0.303				GDP	0.535	3.165	0.005
7	0.167	1.910	0.175	Temp	0.282	1.170	0.257	0.251	3.18	0.064	Temp	0.18	0.787	0.441
				GDP	−0.187	−0.774	0.448				GDP	−0.386	−1.685	0.108
8	0.750	28.515	0.000	Temp	0.105	0.789	0.440	0.652	17.819	0	Temp	−0.163	−1.04	0.312
				GDP	0.809	6.092	0.000				GDP	0.877	5.602	0
9	0.285	3.593	0.049	Temp	0.240	1.199	0.246	0.11	1.117	0.349	Temp	0.234	1.047	0.309
				GDP	−0.458	−2.288	0.034				GDP	−0.217	−0.975	0.343
10	0.977	400.259	0.000	Temp	−0.011	−0.319	0.754	0.991	1065.496	0	Temp	−0.002	−0.068	0.946
				GDP	0.991	27.635	0.000				GDP	0.996	44.989	0
11	0.160	1.815	0.190	Temp	0.399	1.813	0.086	0.154	1.733	0.203	Temp	0.39	1.766	0.093
				GDP	−0.005	−0.023	0.982				GDP	−0.009	−0.039	0.969
12	0.637	14.892	0.000	Temp	0.557	3.554	0.002	0.647	15.6	0	Temp	0.554	3.582	0.002
				GDP	0.404	2.574	0.020				GDP	0.417	2.699	0.015
13	0.846	52.285	0	Temp				0.846	52.285	0	Temp	0.314	3.085	0.006
				GDP							GDP	0.731	7.187	0
14	0.757	29.543	0.000	Temp	0.240	1.880	0.076	0.172	1.979	0.166	Temp	0.464	1.967	0.064
				GDP	−0.732	−5.721	0.000				GDP	0.278	1.18	0.253
15	0.946	165.832	0	Temp				0.946	165.832	0	Temp	0.034	0.633	0.534
				GDP							GDP	0.966	17.863	0
16	0.303	4.129	0.032	Temp	0.443	2.220	0.039	0.514	10.044	0.001	Temp	0.348	2.09	0.05
				GDP	−0.225	−1.129	0.273				GDP	−0.536	−3.219	0.005
17	0.767	31.188	0.000	Temp	0.140	1.081	0.293	0.858	57.23	0	Temp	0.139	1.374	0.185
				GDP	0.794	6.111	0.000				GDP	0.846	8.336	0

Green highlights indicate statistically significant results (*p* < 0.05).

**Table 3:** The correlation between CCP/CCD and TEMP for each country.

Country	Cervical cancer prevalence (CCP)							Cervical cancer death (CCD)						
	R square	F	*p*	independent variable	Beta	*t*	*p*	R square	F	*p*	independent variable	Beta	*t*	*p*
1	0.818	40.334	0.000	Temp	−0.028	−0.274	0.787	0.899	84.635	0.000	Temp	−0.017	−0.235	0.816
				GDP	0.905	8.980	0.000				GDP	0.950	12.956	0.000
2	0.601	14.281	0.000	Temp	0.382	2.488	0.022	0.419	6.847	0.006	Temp	0.210	1.131	0.272
				GDP	0.559	3.637	0.002				GDP	0.547	2.949	0.008
3	0.816	42.211	0.000	Temp	0.067	0.562	0.581	0.959	224.143	0.000	Temp	−0.001	−0.010	0.992
				GDP	0.864	7.249	0.000				GDP	0.980	17.474	0.000
4	0.354	5.215	0.016	Temp	0.370	1.990	0.061	0.654	17.938	0.000	Temp	0.300	2.204	0.040
				GDP	0.421	2.265	0.035				GDP	0.713	5.237	0.000
5	0.641	16.941	0.000	Temp	0.081	0.536	0.598	0.687	20.888	0.000	Temp	0.141	0.999	0.330
				GDP	0.763	5.043	0.000				GDP	0.760	5.385	0.000
6	0.434	7.291	0.004	Temp	−0.138	−0.802	0.432	0.453	7.865	0.003	Temp	0.449	2.645	0.016
				GDP	−0.645	−3.740	0.001				GDP	0.505	2.978	0.008
7	0.190	2.228	0.135	Temp	−0.139	−0.585	0.565	0.167	1.905	0.176	Temp	0.169	0.699	0.493
				GDP	0.349	1.467	0.159				GDP	−0.297	−1.231	0.233
8	0.679	20.095	0.000	Temp	0.298	1.982	0.062	0.777	33.098	0.000	Temp	−0.106	−0.849	0.407
				GDP	0.633	4.204	0.000				GDP	−0.823	−6.564	0.000
9	0.235	2.768	0.090	Temp	0.257	1.242	0.230	0.060	0.570	0.575	Temp	0.244	1.066	0.301
				GDP	−0.390	−1.887	0.075				GDP	0.006	0.025	0.980
10	0.996	2265.057	0.000	Temp	−0.015	−0.996	0.332	0.992	1121.518	0.000	Temp	0.001	0.024	0.981
				GDP	1.001	65.789	0.000				GDP	0.996	46.135	0.000
11	0.335	4.793	0.021	Temp	0.240	1.227	0.235	0.283	3.747	0.042	Temp	0.237	1.167	0.258
				GDP	0.603	3.078	0.006				GDP	0.551	2.711	0.014
12	0.467	7.452	0.005	Temp	0.505	2.658	0.017	0.624	14.093	0.000	Temp	0.605	3.790	0.001
				GDP	0.313	1.647	0.118				GDP	0.334	2.095	0.051
13	0.799	37.685	0.000	Temp	0.243	2.084	0.051	0.729	25.543	0.000	Temp	0.199	1.477	0.156
				GDP	0.754	6.484	0.000				GDP	0.742	5.498	0.000
14	0.820	43.419	0.000	Temp	0.254	2.309	0.032	0.430	7.167	0.005	Temp	0.185	0.943	0.357
				GDP	−0.759	−6.915	0.000				GDP	0.721	3.684	0.002
15	0.878	68.142	0.000	Temp	0.070	0.862	0.400	0.013	0.126	0.883	Temp	−0.043	−0.185	0.855
				GDP	0.923	11.353	0.000				GDP	−0.099	−0.430	0.672
16	0.236	2.941	0.077	Temp	0.472	2.261	0.036	0.503	9.605	0.001	Temp	0.344	2.042	0.055
				GDP	−0.044	−0.209	0.837				GDP	−0.531	−3.150	0.005
17	0.612	14.982	0.000	Temp	0.153	0.913	0.373	0.832	47.031	0.000	Temp	0.159	1.441	0.166
				GDP	0.692	4.129	0.001				GDP	0.819	7.432	0.000

Green highlights indicate statistically significant results (*p* < 0.05).

**Table 4:** The correlation between OCP/OCD and TEMP for each country.

Country	Ovarian cancer prevalence (OCP)							Ovarian cancer death (OCD)						
	R square	F	*p*	independent variable	Beta	T	*p*	R Square	F	*p*	independent variable	Beta	t	*p*
1	0.888	75.584	0.000	Temp	−0.022	−0.288	0.776	0.938	144.191	0.000	Temp	−0.007	−0.119	0.907
				GDP	0.945	12.250	0.000				GDP	0.969	16.893	0.000
2	0.799	37.817	0.000	Temp	0.390	3.583	0.002	0.775	32.755	0.000	Temp	0.334	2.893	0.009
				GDP	0.685	6.287	0.000				GDP	0.712	6.171	0.000
3	0.919	108.324	0.000	Temp	−0.035	−0.445	0.661	0.955	200.663	0.000	Temp	−0.078	−1.316	0.204
				GDP	0.978	12.389	0.000				GDP	1.019	17.236	0.000
4	0.662	18.596	0.000	Temp	0.267	1.981	0.062	0.721	24.516	0.000	Temp	0.249	2.033	0.056
				GDP	0.735	5.463	0.000				GDP	0.780	6.381	0.000
5	0.636	16.611	0.000	Temp	0.097	0.636	0.532	0.664	18.781	0.000	Temp	0.129	0.881	0.389
				GDP	0.752	4.939	0.000				GDP	0.753	5.143	0.000
6	0.273	3.570	0.048	Temp	0.457	2.334	0.031	0.406	6.488	0.007	Temp	0.477	2.697	0.014
				GDP	0.258	1.320	0.202				GDP	0.426	2.411	0.026
7	0.504	9.636	0.001	Temp	0.074	0.398	0.695	0.468	8.340	0.003	Temp	0.138	0.715	0.483
				GDP	−0.670	−3.592	0.002				GDP	−0.604	−3.130	0.006
8	0.726	25.215	0.000	Temp	0.154	1.111	0.281	0.706	22.789	0.000	Temp	0.054	0.376	0.711
				GDP	0.764	5.501	0.000				GDP	0.812	5.634	0.000
9	0.259	3.145	0.067	Temp	0.235	1.156	0.263	0.102	1.017	0.382	Temp	0.221	0.985	0.338
				GDP	−0.432	−2.121	0.048				GDP	−0.212	−0.945	0.357
10	0.974	356.596	0.000	Temp	−0.014	−0.376	0.711	0.973	341.573	0.000	Temp	−0.008	−0.198	0.845
				GDP	0.990	26.100	0.000				GDP	0.988	25.508	0.000
11	0.220	2.674	0.095	Temp	0.400	1.883	0.075	0.268	3.483	0.051	Temp	−0.007	−0.119	0.907
				GDP	−0.154	−0.724	0.478				GDP	0.969	16.893	0.000
12	0.606	13.058	0.000	Temp	0.542	3.317	0.004	0.619	13.821	0.000	Temp	0.334	2.893	0.009
				GDP	0.396	2.423	0.027				GDP	0.712	6.171	0.000
13	0.837	48.960	0.000	Temp	0.288	2.754	0.013	0.836	48.525	0.000	Temp	−0.078	−1.316	0.204
				GDP	0.745	7.124	0.000				GDP	1.019	17.236	0.000
14	0.757	29.557	0.000	Temp	0.262	2.047	0.055	0.154	1.735	0.203	Temp	0.249	2.033	0.056
				GDP	−0.717	−5.606	0.000				GDP	0.780	6.381	0.000
15	0.872	64.915	0.000	Temp	0.051	0.614	0.547	0.879	68.723	0.000	Temp	0.129	0.881	0.389
				GDP	0.924	11.132	0.000				GDP	0.753	5.143	0.000
16	0.386	5.971	0.010	Temp	0.421	2.245	0.037	0.533	10.832	0.001	Temp	0.477	2.697	0.014
				GDP	−0.354	−1.892	0.074				GDP	0.426	2.411	0.026
17	0.767	31.297	0.000	Temp	0.141	1.088	0.290	0.834	47.888	0.000	Temp	0.138	0.715	0.483
				GDP	0.794	6.119	0.000				GDP	−0.604	−3.130	0.006

Green highlights indicate statistically significant results (*p* < 0.05).

**Table 5:** The correlation between UCP/UCD and TEMP for each country.

Country	Ovarian cancer prevalence (UCP)							Uterine cancer death (UCD)						
	R square	F	*p*	independent variable	Beta	*t*	*p*	R square	F	*p*	independent variable	Beta	*t*	*p*
1	0.806	39.489	0.000	Temp	−0.043	−0.419	0.680	0.913	99.592	0.000	Temp	−0.014	−0.206	0.839
				GDP	0.901	8.870	0.000				GDP	0.957	14.050	0.000
2	0.494	9.259	0.002	Temp	0.135	0.781	0.445	0.570	12.587	0.000	Temp	0.242	1.518	0.146
				GDP	0.646	3.733	0.001				GDP	0.639	4.008	0.001
3	0.959	223.304	0.000	Temp	0.021	0.377	0.711	0.909	95.180	0.000	Temp	−0.015	−0.183	0.857
				GDP	0.967	17.220	0.000				GDP	0.962	11.485	0.000
4	0.623	15.712	0.000	Temp	0.281	1.976	0.063	0.466	8.287	0.003	Temp	0.326	1.927	0.069
				GDP	0.702	4.946	0.000				GDP	0.559	3.306	0.004
5	0.611	14.903	0.000	Temp	0.110	0.697	0.494	0.689	21.083	0.000	Temp	0.113	0.804	0.432
				GDP	0.729	4.629	0.000				GDP	0.777	5.519	0.000
6	0.340	4.891	0.019	Temp	0.484	2.597	0.018	0.361	5.367	0.014	Temp	0.437	2.384	0.028
				GDP	0.329	1.766	0.094				GDP	0.416	2.268	0.035
7	0.051	0.505	0.611	Temp	0.254	0.986	0.336	0.120	1.300	0.296	Temp	0.220	0.888	0.386
				GDP	0.083	0.322	0.751				GDP	−0.180	−0.725	0.477
8	0.598	14.139	0.000	Temp	0.246	1.460	0.161	0.500	9.497	0.001	Temp	−0.153	−0.816	0.425
				GDP	0.620	3.682	0.002				GDP	0.772	4.110	0.001
9	0.083	0.813	0.459	Temp	0.214	0.946	0.357	0.063	0.601	0.559	Temp	0.239	1.042	0.311
				GDP	−0.175	−0.773	0.450				GDP	−0.057	−0.250	0.805
10	0.770	31.777	0.000	Temp	−0.003	−0.023	0.982	0.988	808.266	0.000	Temp	−0.005	−0.215	0.832
				GDP	0.878	7.772	0.000				GDP	0.995	39.218	0.000
11	0.301	4.082	0.034	Temp	0.408	2.029	0.057	0.132	1.441	0.261	Temp	0.375	1.674	0.111
				GDP	−0.265	−1.320	0.202				GDP	0.172	0.769	0.451
12	0.575	11.478	0.001	Temp	0.622	3.664	0.002	0.736	23.675	0.000	Temp	0.434	3.246	0.005
				GDP	0.263	1.552	0.139				GDP	0.599	4.481	0.000
13	0.829	45.986	0.000	Temp	0.357	3.330	0.004	0.766	31.013	0.000	Temp	0.226	1.803	0.087
				GDP	0.687	6.404	0.000				GDP	0.746	5.942	0.000
14	0.737	26.557	0.000	Temp	0.139	1.042	0.311	0.329	4.668	0.022	Temp	0.404	1.905	0.072
				GDP	−0.785	−5.898	0.000				GDP	0.637	3.001	0.007
15	0.732	26.013	0.000	Temp	0.041	0.338	0.739	0.871	64.248	0.000	Temp	0.078	0.938	0.360
				GDP	0.848	7.057	0.000				GDP	0.918	11.000	0.000
16	0.465	8.243	0.003	Temp	0.366	2.094	0.050	0.567	12.440	0.000	Temp	0.325	2.063	0.053
				GDP	−0.481	−2.751	0.013				GDP	−0.595	−3.780	0.001
17	0.773	32.419	0.000	Temp	0.129	1.009	0.326	0.846	52.238	0.000	Temp	0.148	1.407	0.176
				GDP	0.805	6.290	0.000				GDP	0.834	7.905	0.000

Green highlights indicate statistically significant results (*p* < 0.05).

### Variables

Dependent variables:

1. Breast cancer prevalence—percentage (BCP)
2. Cervical cancer prevalence—percentage (CCP)
3. Ovarian cancer prevalence—percentage (OCP)
4. Uterine cancer prevalence—percentage (UCP)
5. Breast cancer deaths—percentage (BCD)
6. Cervical cancer deaths—percentage (CCD)
7. Ovarian cancer deaths—percentage (OCD)
8. Uterine cancer deaths—percentage (UCD)

Independent variable: Temperature change with respect to a baseline climatology (TEMP).

Controlling variable: Gross Domestic Product per capita (GDP).

Data for all these variables was collected for the years 1998 till 2019.

### Data sources

Secondary data was used in this research. Datasets were derived from three main sources:

1. Data for the eight dependent variables on the prevalence and deaths caused by cancer were extracted from the Global Burden of Disease data provided by the Institute of Health Metrics and Evaluation (IHME) (26).
2. Data on temperature change was obtained from the Food and Agriculture Organization of the United Nations (FAO) – FAOSTAT Climate Change database (27).
3. GDP per capita data was derived from the World Bank Databank (28).

### Sample selection

The countries included in the study were selected based on two criteria: geographic representation (i.e., part of the MENA region according to the World Bank classification) and the availability of data. Taking into account these two criteria the study ended up with 17 countries. The countries included are: 1 = Algeria, 2 = Bahrain, 3 = Egypt, 4 = Iran, 5 = Iraq, 6 = Jordan, 7 = Kuwait, 8 = Lebanon, 9 = Libya, 10 = Morocco, 11 = Oman, 12 = Qatar, 13 = Saudi Arabia, 14 = Syria, 15 = Tunisia, 16 = United Arab Emirates (UAE), and 17 = Palestine.

### Ethics and consent

The study did not involve human participants, materials, or data; therefore, no institutional review board, ethics committee approval, or consent was required.

### Multiple linear regression

MLR models the relationship between each dependent variable and temperature, controlling for GDP. MLR was performed on all countries combined and then separately for each country. A significance cutoff of *p* < 0.05 was used.

The MLR model:


$$y=\beta_{0}+\beta_{1}{Χ}_{1}+\beta_{2}{Χ}_{2}+\in_{i}$$

y : dependent variable (BCP, CCP, OCP, UCP, BCD, CCD, OCD, UCD).

$\beta_{0}$ : intercept.

$\beta_{1}$ : regression coefficients TEMP.

$X_{1}:$ values of TEMP for country i.

$\beta_{2}$ : regression coefficients GDP

$X_{2}:$ values of GDP for country i

$\in_{i}:$ error term

Analysis was conducted using SPSS version 26.

Multicollinearity was assessed. The Variance Inflation Factor (VIF) test was conducted, and the results showed that all independent variables had VIF values below 5 (Temperature Change: VIF = 1.015, GDP per Capita: VIF = 1.015), indicating no multicollinearity issues.

## Results

The results of this study are two parts, regional and country level. This first part covers the MLR analysis investigating the relation between the prevalence and deaths from four types of women’s cancer and the independent variable TEMP while controlling for GDP in the MENA region. The results (Table 1) showed that seven of the eight dependent variables were statistically significant (*p* < 0.05), indicating that the models effectively explain the variance in cancer deaths and the prevalence of breast, ovarian, and cervical cancers.

For every one-unit increase in TEMP, deaths from the four cancers increased by 0.171 to 0.332 units, with ovarian cancer showing the highest correlation (0.332) and cervical cancer the lowest (0.171). Regarding prevalence, the B coefficient showed an increase between 0.173 and 0.280 units, with ovarian cancer having the highest correlation and breast cancer the lowest.

The second part examines MLR results for each of the 17 countries.

### Breast cancer

Significant positive correlations were found between BCP and TEMP in Bahrain, Qatar, and UAE, with a one-degree increase in TEMP resulting in 0.33, 0.56, and 0.44 increases in BCP, respectively. BCD also showed significant correlations in Jordan, Qatar, Saudi Arabia, and UAE, with increases of 0.42, 0.55, 0.31, and 0.35, respectively, per degree increase in TEMP (Table 2).

### Cervical cancer

There were significant positive correlations between CCP and TEMP in Bahrain, Qatar, and Syria, with increases of 0.38, 0.51, and 0.25, respectively, for each degree increase. CCD was significantly correlated with TEMP in Iran, Jordan, and Qatar, with increases of 0.3, 0.45, and 0.61, respectively (Table 3).

### Ovarian cancer

OCP correlated significantly with TEMP in Bahrain, Jordan, Qatar, Saudi Arabia, and UAE, with increases of 0.39, 0.46, 0.54, 0.29, and 0.421, respectively. OCD correlated significantly with TEMP in Bahrain, Jordan, Qatar, and UAE, with changes of 0.33, 0.48, 0.33, and 0.48, respectively (Table 4).

### Uterine cancer

UCP was significantly correlated with TEMP in Jordan, Qatar, Saudi Arabia, and UAE, with increases of 0.48, 0.62, 0.36, and 0.37, respectively. UCD showed significant correlations with TEMP in Jordan and Qatar, increasing by 0.44 and 0.43, respectively, per degree increase (Table 5).

## Discussion

As indicated above, MENA region is a climate change high risk area characterized by temperature average increase that is above the average for other regions. The results of this study show that climate change represented by increased temperature is significantly related to increased deaths and prevalence of cancer in women in the MENA region.

Several individual and socioeconomic factors may increase the risk of cancer in women (29). Climate changes including temperature are found to affect the social determinants of cancer including availability and quality of clean air, water and housing. As well as its effect on cancer health care infrastructure and the accessibility to these infrastructures in countries (13). Prolonged ambient high-temperature exposure is not yet recognized as one of these risk factors. There is no identified direct relation between increased atmospheric temperature prolonged exposure and cancer prevalence and deaths, the effect of climate change on the process of carcinogenesis is not proven yet. Nevertheless, prolonged exposure to a combination of rising ambient temperatures and traffic-related air pollution has been associated with an increased risk of gynecological cancers, including cervical, endometrial, and ovarian cancers (30).

Moreover, research suggests that external temperature influences the mechanical properties of cells. Even minor temperature changes can significantly alter cell characteristics, with increased temperatures enhancing optical deformability, including in breast cells (31, 32).

**Figure 1:**
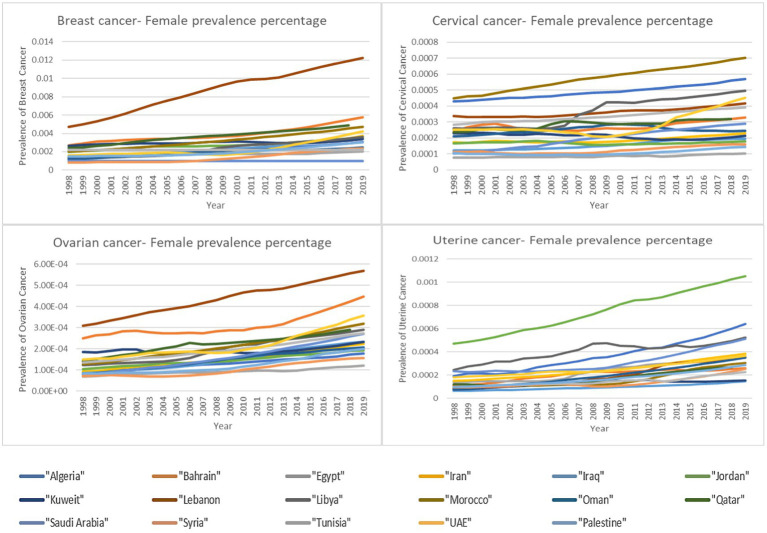
Percentage of female deaths by cancer type over time.

This study concentrated on two measures for each type of women’s cancer included: mortality (deaths) and prevalence. Mortality rates reflect the incidence of the disease as well as the availability of early detection and treatment. Cancer is one of the main causes of mortality among women around the world (37). Given the general improvements in cancer treatment and early diagnosis (33, 34), the mortality rates of cancers in women ought to have decreased over time. Nevertheless, looking at the data for 17 countries in the MENA region (Figure 1), it is observed that this was not the case for the four types of cancers in women in these countries. Indicating that incidence rate effect has exceeded the effect of early detection and treatment and according to this study results, incidence is correlated to the increase in ambient temperature.

**Figure 2:**
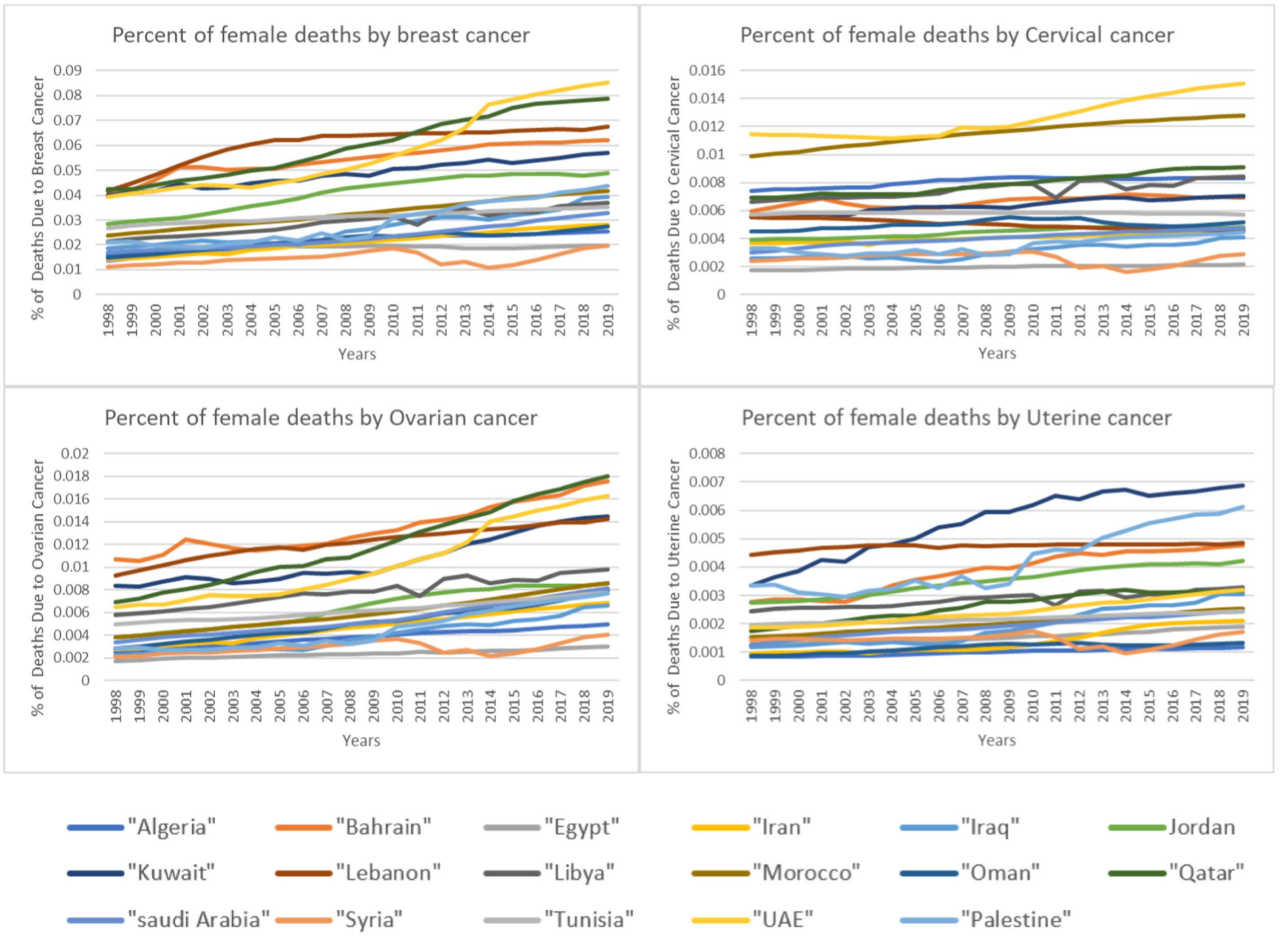
Percentage of female deaths by cancer type over time.

As for the prevalence of a disease, it reflects both the exposure to risk factors and the availability of screening. Advancement in cancer screening methods over the years contributes to the increased prevalence as more cases are discovered through screening. However, this does not annulated the contribution of increased exposure to risk factors. Figure 2 provides an overview of the prevalence of the four types of women’s cancer in the 17 countries included in the study demonstrating the general trend in increased prevalence of these cancers among women in these 17 countries over the years.

Screening and treatment are factors related to the level of medical advancement in a country as well as on the accessibility to health services. Although there are variations among the 17 countries included in this study in these aspects, these countries have been advancing in terms of medical service availability and accessibility through the years thus the provision of more screening and treatment. Increased screening means increased prevalence but it also means decreased deaths as the increased screening leads to higher probability of treatment and thus less deaths. The fact that both prevalence and deaths increased highlight the importance of the other two factors: exposure to risk factors and subsequently increased incidence. And given the results of this study that prolonged exposure to high ambient temperature is correlated to the prevalence of three women cancers in the MENA region, one can deduct that high ambient temperature can be considered as a potential risk factor.

Although the correlation results for the prevalence and deaths were significant for the region as a whole, the correlation for the disaggregated data by country showed different results. As observed in Figure 1, certain countries exhibit particularly high prevalence rates, standing out as potential outliers. To explore these variations, we examined country-specific trends. The results show that only six countries showed significant correlation between the increase in ambient temperature and the prevalence and/or deaths by the four cancer types (Table 6). Within the six countries, four countries are Gulf countries (Qatar, Bahrain, Saudi Arabia and UAE). Gulf countries are known to have relatively extreme ambient temperatures especially during the summer which could be the reason behind the correlation results. Within these four countries, Qatar was the one with significant correlations for the four cancer types both in prevalence and in deaths indicating the need for further in-depth investigation. In addition to these four gulf countries, Jordan shows similar results which raises further questions. According to Jordan’s Climate Risk Profile (35), the country is facing drastic effects of climate change manifested in extreme weather events such as floods, droughts and increased ambient temperature. The projection estimates that by 2080 air temperatures in Jordan will rise by up to 4.5°C. Furthermore, there is a high degree of certainty that the number of days per year in Jordan with a maximum temperature above 35°C will increase. These climate changes effects in Jordan might be the reason behind the significant correlation between the increase in ambient temperature and the prevalence of two women’s cancers and the deaths caused by all four types of cancers. Lastly, within the significant correlation results, Syria had a significant correlation between increased ambient temperature and the prevalence of cervical cancer while Iran had a significant correlation with cervical cancer deaths. These two occurrences can be related to increased temperature where Iranian population is subjected to periods of prolonged heat especially in urban areas (36) and Syria is experiencing increases in temperatures. Although in the case of Syria, temperature increase is not that extreme in populated areas which leads to the conclusion that other factors might be contributing to this result.

**Table 6:** Significant correlation values for disintegrated data by country on the prevalence of the four cancer types*.

	Qatar		UAE		Bahrain		Jordan		Saudi Arabia	
	P	D	P	D	P	D	P	D	P	D
Breast cancer	0.56	0.55	0.44	0.35	0.33			0.42		0.31
Cervical cancer	0.51	0.61			0.38			0.45		
Ovarian cancer	0.54	0.33	0.42	0.48	0.39	0.33	0.46	0.48	0.29	
Uterine cancer	0.62	0.43	0.366				0.48	0.44	0.36	

P, Prevalence; D, Death. *This table summarizes only the statistically significant associations identified in Tables 2–5. All corresponding *p*-values are reported in full in those tables. Significant values here are highlighted in green for visual emphasis.

Some countries, such as Algeria and Lebanon, did not exhibit statistically significant associations between ambient temperature and cancer outcomes across the four cancer types. These non-significant results may reflect different local contexts, milder temperature variations, or other dominant health determinants not captured in the current model.

Although more research is needed to validate and generalize the findings of this study, the findings highlight the necessity of including climate change adaptation measures into national cancer control plans. This can be achieved by encouraging interdisciplinary methods to address the confluence of climate and health, promoting sustainable environmental policies to limit the implications of climate change, and including climate adaption strategies into the planning of health care infrastructure.

### Study limitations

While this study provides significant insights, it is considered preliminary research due to several factors including: limited studies specifically focusing on the relation between increased ambient temperature and cancer or the mechanism involved. The correlation between temperature, cancer prevalence, and death does not imply causation. There can be other contributing factors including genetics, lifestyle, exposure to environmental pollutants (e.g., PFAS), and access to health care, gender disparities, long-term exposure to carcinogens such as PM2.5 and endocrine-disrupting chemicals. Additionally, differences in heat acclimatization were not considered, which may influence how populations in warmer climates respond to rising temperatures.

It is difficult to separate the precise effect of temperature from these other variables. Further research is required to investigate the underlying mechanisms and any confounding variables that can explain the observed connections between temperature, cancer, and GDP in the MENA region. Finally, the study’s scope is limited to a small number of MENA countries, which may affect the generalizability of findings. Expanding the analysis to other regions could help determine whether similar patterns exist globally.

## Conclusion

This study highlights how climate change is no longer a distant environmental concern but a pressing threat to women’s health. The findings support the existence of a correlation between prolonged exposure to high temperature and the burden of women’s cancers in the MENA region. The relationship was evident at both the regional and country levels and was more pronounced in countries experiencing extreme heat.

The implications of these findings are important. It calls for immediate attention from policymakers and health planners. It highlights the urgency of integrating climate-related risks into health policy, with a focus on women’s health. Countries with high exposure should strengthen their cancer early detection and response systems. This includes improving awareness, screening, and access to care. Cross-sectoral collaboration between health, environment, and planning institutions is needed. Countries with lower burden should also act early, using these findings as an early warning.

The study adds to the growing recognition of the intersection between climate and health. It contributes to shaping future dialog and action on equitable, climate-resilient health systems in the region. Nevertheless, the study has limitations. Further work is needed to validate the findings using larger samples and to explore the mechanisms linking temperature and cancer.

Finally, this research’s results call for coordinated, climate-informed public health policies that protect vulnerable populations—especially women—from compounding environmental risks.

## Data Availability

The raw data supporting the conclusions of this article will be made available by the authors, without undue reservation.
